# Active Rectal Bleeding Due to Polyp Avulsion Induced by Bowel Preparation: A Case Report

**DOI:** 10.7759/cureus.92139

**Published:** 2025-09-12

**Authors:** Jiaming Lei, Ling Wu

**Affiliations:** 1 Department of Gastroenterology, People’s Hospital of Leshan, Leshan, CHN; 2 Department of Cardiology, Affiliated Hospital of Southwest Medical University, Luzhou, CHN

**Keywords:** chicken skin mucosa (csm), endoscopy, hemostasis, intestinal polyp, lower gastrointestinal bleeding

## Abstract

Lower gastrointestinal bleeding (LGIB) represents a significant clinical burden. While typically attributed to diverticulosis or angiodysplasia, iatrogenic mechanisms remain poorly characterized. This report describes a case of active rectal bleeding in a 32-year-old male patient following bowel preparation for colonoscopy. Endoscopy revealed massive fresh red blood and clots in the rectum, with an 8-mm mucosal protrusion showing arterial pulsatile bleeding at 8 cm from the anal verge. Characteristic chicken skin mucosa (CSM) surrounded the lesion. Based on the hemorrhagic lesion morphology and CSM features, it was postulated as a mechanical avulsion of a pedunculated (Ip-type) polyp induced by friction from hardened stool or a fiber-induced "snare-like" effect during bowel preparation, exposing vessels and causing massive hemorrhage. This case provides a novel perspective on "bowel preparation-induced polyp avulsion" as a mechanism for LGIB and highlights the potential of CSM as a marker for underlying neoplastic risk.

## Introduction

Lower gastrointestinal bleeding (LGIB) remains a diagnostic challenge despite endoscopic advancements, with 10-15% of cases classified as obscure hemorrhages [1]. The incidence increases with age, affecting 200 of 100,000 individuals aged over 80 years annually [2]. Current epidemiological data indicate that the prevalence of adenoma among people of screening age is 20-60%, and it increases with age. The incidence of adenoma is relatively balanced across all intestinal segments (except that in early-onset individuals under 50 years old, it is more frequently seen in the distal colorectum). Bowel preparation introduces unique physiological stressors, including altered colonic motility and intraluminal pressure changes that may precipitate complications. Common adverse events related to intestinal preparation include electrolyte imbalance, mucosal irritation, and highly rare cases of polyp avulsion injury [3].

The first systematic endoscopic and histologic description of CSM was published in 1998 [4], demonstrating endoscopic yellowish-speckled mucosa corresponding histologically to lamina propria lipid-laden macrophages. This marker identifies patients with a 4.7-fold increased risk of colorectal adenomas and a 9.2-fold higher likelihood of advanced neoplasia [5]. We present a case of acute massive LGIB post bowel preparation in which CSM provided critical diagnostic insights, highlighting both mechanical complications of purgative use and CSM's role in neoplasia risk stratification.

## Case presentation

A 32-year-old male (height 175 cm, weight 70 kg, BMI 26.67 kg/m²) with a history of chronic constipation (defecation frequency once every four days) presented for elective colonoscopy. The indication for colonoscopy was evaluation of chronic constipation and routine screening. The patient underwent bowel preparation using polyethylene glycol electrolyte powder (137.15 g dissolved in 2 L of water), administered as a split-dose regimen completed six hours prior to the procedure.

**Figure 1:**
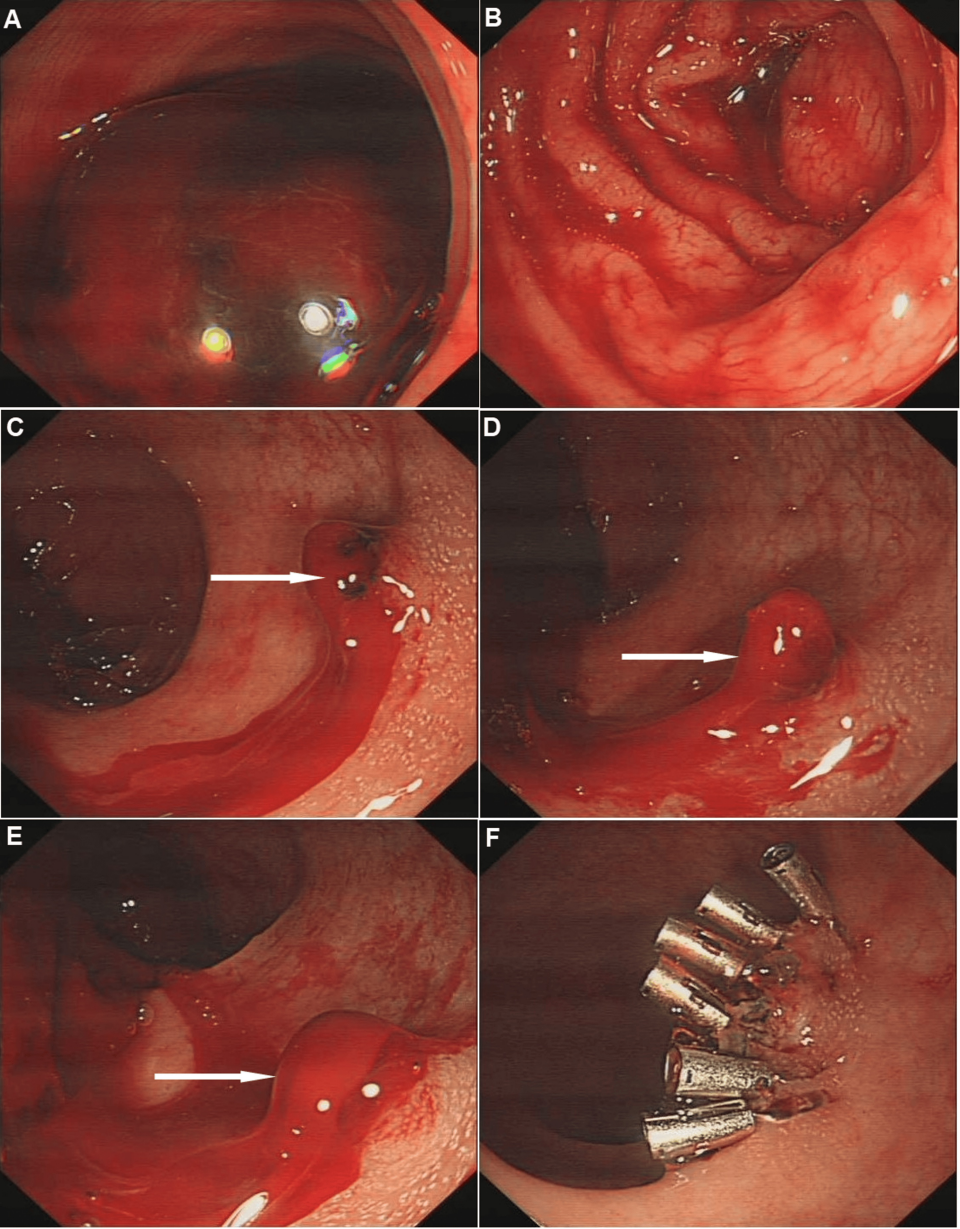
Endoscopic image showing a polyp pedicle-like hemorrhage foci (white arrow) and the CMS mucosa (A) Rectum showing a large amount of blood clot within the lumen; (B) Descending colon with visible fresh blood; (C, D, E) Medium and close-up views of a protruding bleeding lesion on the mucosa, exhibiting arterial pulsation-like oozing; (F) Status of the rectal mucosa post hemostasis. CMS: chicken skin mucosa

Upon colonoscope insertion, massive fresh bright red blood and clots were observed filling the rectal lumen (Figure 1A), with blood reflux visible up to the descending colon (Figure 1B), significantly obscuring visualization. The estimated blood loss was approximately 400-500 ml, based on the volume of blood and clots suctioned during the procedure.

Laboratory tests prior to the procedure showed normal platelet count and coagulation function, with hemoglobin of 135 g/L and hematocrit of 35%. Urgent laboratory tests following colonoscopy revealed a hemoglobin level of 112 g/L and hematocrit of 34%. The patient maintained stable hemodynamics throughout the procedure (blood pressure 125/80 mmHg, heart rate 85 bpm), with no signs of hypovolemia or hemodynamic compromise.

After extensive irrigation and adequate insufflation, an approximately 8-mm mucosal protrusion with active arterial pulsatile bleeding was identified on the anterior rectal wall, 8 cm from the anal verge. The bleeding site was surrounded by white granular mucosa consistent with CSM (Figure 1C-1E).

Given the critical nature of the active bleeding, endoscopic hemostasis was immediately initiated without biopsy to avoid exacerbating hemorrhage. A total of six hemostatic clips were successfully deployed to achieve complete hemostasis (Figure 1F). No medications were administered for bleeding control, and blood transfusion was not required.

Post-procedure history revealed that the patient had passed a large volume of bright red blood during the final evacuation phase of bowel preparation. The patient's recovery was uneventful, and a follow-up colonoscopy at three months showed complete healing with no residual abnormalities.

## Discussion

Common etiologies of LGIB include hemorrhoidal bleeding, malignancy, ischemic colitis, polyp bleeding, diverticular bleeding, inflammatory bowel disease, infectious colitis, Dieulafoy's lesion, vascular malformations/ectasias, radiation proctitis, solitary rectal ulcer, and rectal varices. Recent years have seen increasing contributions from nonsteroidal anti-inflammatory drugs (NSAIDs), aspirin/antiplatelet agents, and anticoagulants [2].

This case of unheralded massive rectal bleeding in a young male is unusual. Although no intact polyp was visualized, the lesion displayed classic features of a bleeding pedunculated polyp stump (wick-like appearance, arterial spurting), combined with the characteristic surrounding white granular CSM [3]. This strongly suggests spontaneous avulsion of a rectal Ip-type polyp. While polyps themselves are prone to bleeding (correlated with size, rich vasculature, friability, inflammation, stool friction, surface erosion, or malignant invasion), this young patient's small lesion caused disproportionately massive, torrential hemorrhage.

The hypothesis is that chronic constipation led to hardened stool accumulating in the rectum. During vigorous defecation induced by purgatives, friction between the adherent stool and the polyp apex, concentrated at its weakest point (the stalk), caused mechanical avulsion of the pedunculated polyp. We also hypothesize that undigested fibrous materials or hair-like strands within the stool might have acted like a snare, contributing to the avulsion. Exposed vessels in the thick stalk resulted in arterial bleeding, compounded by the flushing action of bowel preparation fluid, preventing clot formation and spontaneous hemostasis. Therefore, pre-procedure instructions should emphasize a low-residue diet, avoiding high-fiber vegetables to reduce risks of obstructive complications or similar avulsion events.

Notably, CSM served as a significant clue in this case. CSM, characterized histologically by lipid-laden macrophage aggregates in the lamina propria, is associated with colorectal adenomas (present in 30.7% based on studies) [1]. It correlates significantly with younger patient age, 32 years in the present case, multiplicity of adenomas (≥2), polypoid morphology, and risk of advanced neoplasia (high-grade dysplasia/villous components) [5]. Its distribution is predominantly distal (93.3% in rectum/sigmoid, matching the lesion site in the current case), potentially linked to a pro-tumorigenic chronic inflammatory microenvironment mediated by dense macrophage infiltration [4]. Although no residual neoplastic tissue was detected, the presence of CSM suggests this site may have harbored an advanced adenoma [6]. Vigilance for local recurrence or synchronous/metachronous neoplasms is warranted.

## Conclusions

This case highlights bowel preparation as a potential trigger for mechanical avulsion of pedunculated colorectal polyps, resulting in life-threatening arterial bleeding. The hypothesis of physical disruption by hardened stool or fiber-induced traction during purging warrants careful consideration in constipated patients undergoing colonoscopy. The presence of CSM adjacent to the bleeding site offers an important endoscopic clue suggesting localized neoplastic change, even in the absence of a detectable polyp. Although this association remains speculative without histologic confirmation of the avulsed lesion, this case underscores the need for heightened clinical vigilance regarding bowel preparation-related complications. Further clinical studies are essential to validate CSM as a predictive marker and to establish evidence-based strategies for risk mitigation and timely endoscopic management in such scenarios.
